# Giant Right Atrial Myxoma Coexisting With Tricuspid Regurgitation, Coronary Artery Disease, and Aortic Stenosis

**DOI:** 10.7759/cureus.75311

**Published:** 2024-12-08

**Authors:** Baku Takahashi, Pirapon Momsila, Hideki Isa, Nuttapon Arayawudhikul

**Affiliations:** 1 Cardiovascular and Thoracic Unit, Department of Surgery, Lampang Hospital, Lampang, THA; 2 Cardiovascular Thoracic Surgery Unit, Department of Surgery, Chiang Mai University, Chiang Mai, THA

**Keywords:** aortic stenosis, cardiac tumor, coronary artery disease, right atrial myxoma, tricuspid regurgitation

## Abstract

A 70-year-old man presented to our hospital with chest discomfort and epigastric pain. Echocardiography revealed a giant atrial myxoma in the right atrium with severe tricuspid regurgitation. The aortic valve was calcified, and severe aortic stenosis was observed. Coronary angiography revealed diffuse lesions in the right coronary artery. The myxoma was surgically excised, and the residual septal defect was repaired using autologous pericardium tissue. Tricuspid valve annuloplasty, aortic valve replacement, and coronary artery bypass grafting were then performed. Clinicians should be aware that myxomas can coexist with other cardiac diseases. Careful preoperative evaluation and surgical planning should be performed prior to urgent surgeries for myxomas.

## Introduction

Myxoma represents the most common type of primary cardiac tumor. Most myxomas occur in the left atrium, whereas right atrial myxomas are relatively rare [1]. Myxomas can coexist with atrial valvular disease depending on their size, location, and mobility [1,2]. However, there are few reports of their copresence with other cardiac diseases. Herein, we report the case of a giant right atrial myxoma coexisting with tricuspid regurgitation (TR), coronary artery disease (CAD), and aortic stenosis (AS). These conditions were identified through meticulous preoperative examinations and treated via concomitant surgical procedures.

## Case presentation

**Figure 1:**
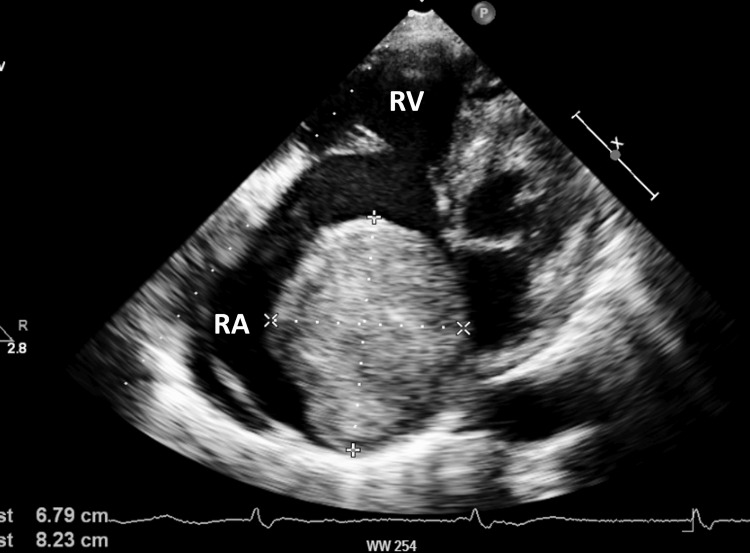
Preoperative echocardiography Preoperative echocardiography shows a large atrial myxoma (82 mm × 68 mm) in the right atrium. RA: Right atrium; RV: Right ventricle.

A 70-year-old man presented with a one-month history of chest discomfort and epigastric pain. He had been taking an antiviral medication for hepatitis B, and had a history of left nephrectomy due to kidney stones. Physical examination revealed a blood pressure of 130/49 mmHg and pulse rate of 60 beats/min. Electrocardiography revealed a normal sinus rhythm with a complete right bundle branch block. Echocardiography revealed a large atrial myxoma (82 × 68 mm) occupying most of the right atrium (Figure 1) and moving toward the tricuspid valve (Video 1).

**Video 1:** Preoperative echocardiography. Preoperative echocardiography shows a giant right myxoma attached to the atrial septum, moving toward the tricuspid valve.

The mass was mobile and pedunculated. There was no coaptation of the tricuspid valve, and severe TR was noted. The aortic valve was heavily calcified, and there was severe AS (aortic valve area = 0.55 cm^2^ by trace, Vmax = 7.3 m/s), although the left ejection fraction was preserved (74% by biplane). Coronary angiography performed to rule out coronary artery disease prior to surgery revealed diffuse lesions in the right coronary artery (75% stenosis at the most severe point). Therefore, surgery was planned to excise the right atrial myxoma and resolve the TR, CAD, and AS.

**Figure 2:**
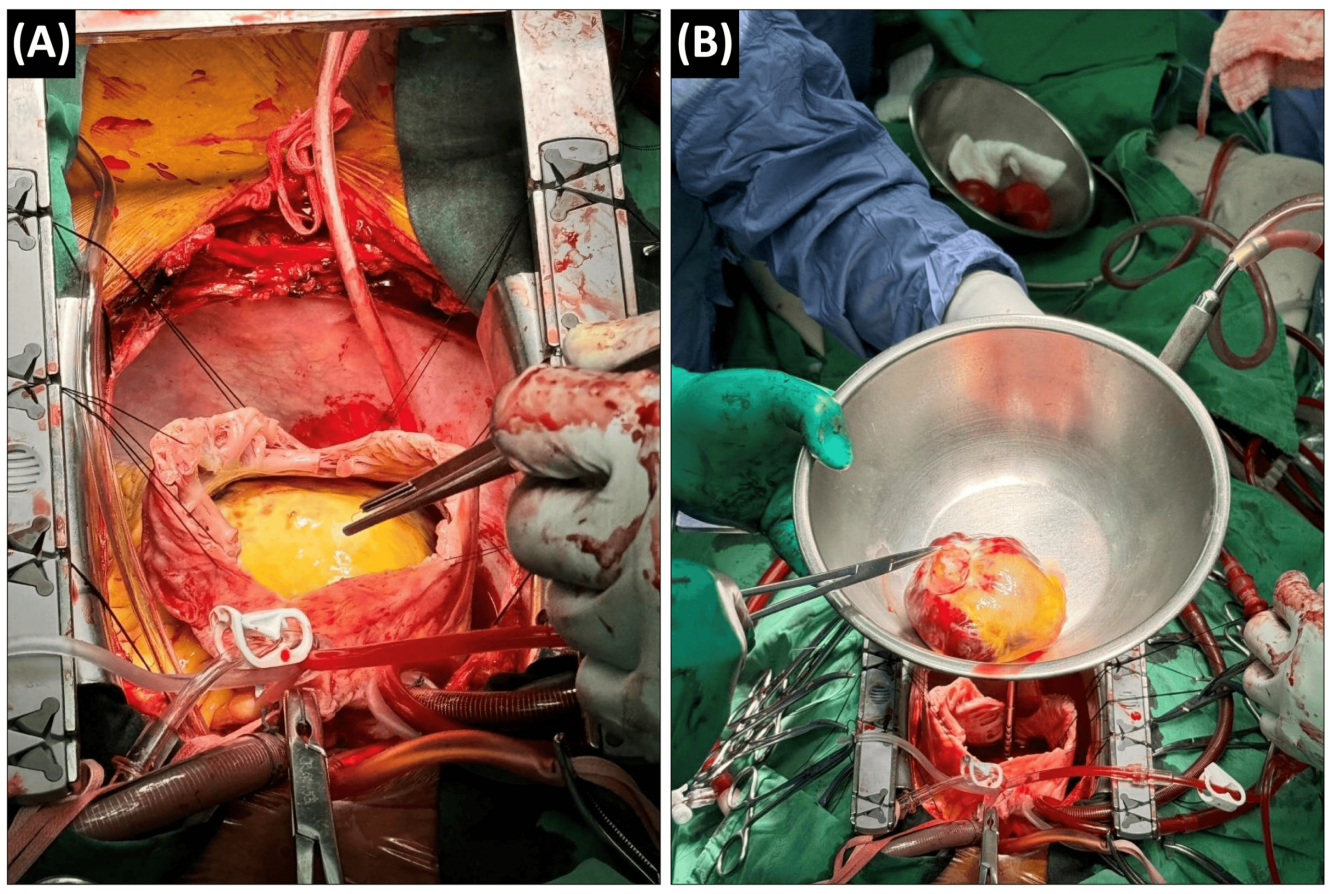
Intraoperative findings. (A) A myxoma occupies most of the right atrium. (B) The excised myxoma is shown.

After median sternotomy, cardiopulmonary bypass (CPB) was established with cannulation in the ascending aorta and venous drainage in the superior vena cava (SVC). The inferior vena cava (IVC) was carefully cannulated through the femoral vein. After cardiac arrest using antegrade coronary perfusion, the right atrium was opened via oblique atriotomy. The tumor almost completely occupied the right atrium (Figure 2A) and was resected along the edge of the atrial septum (Figure 2B).

The residual septal defect was repaired using autologous pericardium tissue. Tricuspid valve annuloplasty was performed using a 32-mm tricuspid annuloplasty ring. The great saphenous vein (SVG) was then bypassed to the distal portion of the right coronary artery. Following aortic valve replacement with a 19-mm tissue valve, proximal anastomosis of the SVG to the ascending aorta was performed. The CPB time was 216 min, and the total cardiac arrest time was 189 min.

**Figure 3:**
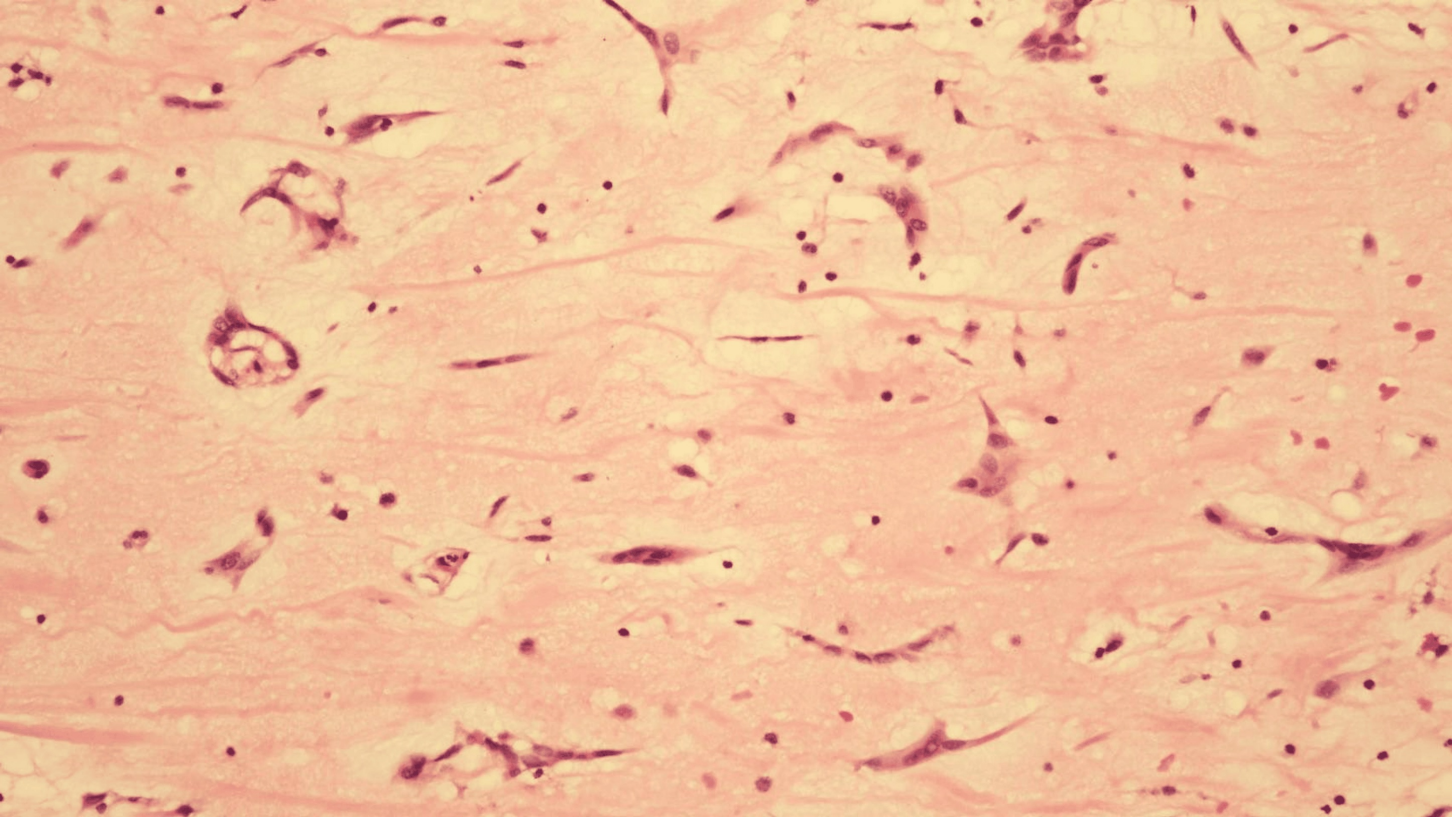
Histopathological image Histopathological examination shows an abundant myxoid stroma with spindle and polygonal tumor cells forming cords (hematoxylin and eosin stain).

No abnormal findings and only mild tricuspid regurgitation were observed on postoperative echocardiography. Histopathological examination revealed a benign myxoma with myxoid stroma (Figure 3).

The postoperative course was uneventful, and the patient was discharged on postoperative day 10.

## Discussion

Cardiac myxomas are the most common heart tumors, with 75% occurring in the left atrium and only 10-15% in the right atrium [1]. Myxomas can cause atrial valvular disease depending on their size, location, and mobility, and multiple cases of right atrial myxoma resulting in TR or pseudo-tricuspid stenosis have been reported [1,2]. However, there is limited information on myxomas co-occurring with other cardiac diseases. To the best of our knowledge, this is the first report of a right atrial myxoma coexisting with TR, CAD, and AS.

According to previous reports, CAD is present in 21-36% of myxoma cases [3,4]. Although unresolved, the not uncommon connection between these diseases may largely reflect their development at similar ages [3,4]. However, because myxomas can cause sudden death, urgent surgery may be undertaken before CAD has been ruled out. The possibility of CAD in patients with myxoma should never be disregarded, and coronary angiography should be performed preoperatively in all myxoma cases if possible [3,4].

In addition to CAD, myxomas can also coexist with AS; in the three cases reported thus far, the myxoma occurred in the left atrium [5,6]. Age-related arteriosclerosis is the most common cause of AS [7], and the patients in two of the three cases were in their 80s; whether they had non-age-related risk factors for arteriosclerosis was unclear.

As the average life expectancy increases worldwide, the number of patients with myxomas coexisting with CAD and AS may also increase. The combination of myxoma and other cardiac diseases may exacerbate heart failure [5], leading to higher perioperative morbidity and mortality rates [6] than those of myxoma alone. Therefore, careful examinations and surgical planning are warranted in older, more vulnerable patient populations.

For both left and right atrial myxomas, bicaval cannulation should effectively improve the operative view and ease the difficulty of the procedure [8]. For right atrial myxomas, SVC cannulation is relatively straightforward to perform, whereas direct cannulation of the IVC without touching the right atrium can be difficult. Therefore, in our case, we inserted an IVC drainage cannula through the femoral vein to avoid possible fragmentation of the myxoma before aortic cross-clamping. When a myxoma is large or highly mobile, the femoral vein is a favorable site for IVC drainage [8].

In patients with myxomas and CAD, the cardiac protection method should be considered. To ensure adequate protection during cross-clamping in these patients, Shiiku et al. recommend performing coronary artery bypass grafting before myxoma resection [9]. However, in our case, doing so was challenging because the myxoma was quite large, making the heart difficult to lift. Although effective [2], retrograde myocardial protection cannot be used in giant right atrial myxoma cases. Therefore, we established CBP and used antegrade coronary perfusion for myocardial protection before resecting the myxoma. Priorities and myocardial protection techniques should be determined according to the myxoma status and the degree, number, and location of the coronary artery stenoses in the patient.

## Conclusions

We describe the copresence of a giant right atrial myxoma and TR, CAD, and AS. Clinicians should be aware that myxomas can coexist alongside other cardiac diseases. Although myxomas require urgent surgery to prevent sudden death, careful preoperative evaluation and surgical planning should not be overlooked. Our experience contributes valuable insights to the treatment strategies for cardiac myxomas and informs future clinical practice.
